# Detection of Critical LUCC Indices and Sensitive Watershed Regions Related to Lake Algal Blooms: A Case Study of Taihu Lake

**DOI:** 10.3390/ijerph120201629

**Published:** 2015-01-29

**Authors:** Chen Lin, Ronghua Ma, Zhihu Su, Qing Zhu

**Affiliations:** 1 State Key Laboratory of Lake Science and Environment, Nanjing Institute of Geography and Limnology, Chinese Academy of Sciences, Nanjing 210008, China; E-Mails: clin@niglas.ac.cn (C.L.); zhsu@163.com (Z.S.); zhuqing@niglas.ac.cn (Q.Z.); 2 State Key Laboratory of Soil and Sustainable Agriculture, Institute of Soil Science, Chinese Academy of Sciences, Nanjing 210008, China

**Keywords:** Land use/cover change (LUCC), landscape, algae area, buffer regions, lake divisions

## Abstract

Taihu Lake in China has suffered from severe eutrophication over the past 20 years which is partly due to significant land use/cover change (LUCC). There is an increasing need to detect the critical watershed region that significantly affects lake water degradation, which has great significance for environmental protection. However, previous studies have obtained conflicting results because of non–uniform lake indicators and inadequate time periods. To identify the sensitive LUCC indices and buffer distance regions, three lake divisions (Meiliang Lake, Zhushan Lake and Western Coastal region) and their watershed region within the Taihu Lake basin were chosen as study sites, the algal area was used as a uniform lake quality indicator and modeled with LUCC indices over the whole time series. Results showed that wetland (WL) and landscape index such as Shannon diversity index (SHDI) appeared to be sensitive LUCC indices when the buffer distance was less than 5 km, while agricultural land (AL) and landscape fragmentation (*Ci*) gradually became sensitive indices as buffer distances increased to more than 5 km. For the relationship between LUCC and lake algal area, LUCC of the WC region seems to have no significant effect on lake water quality. Conversely, LUCC within ML and ZS region influenced algal area of corresponding lake divisions greatly, while the most sensitive regions were found in 3 km to 5 km, rather than the whole catchment. These results will be beneficial for the further understanding of the relationship between LUCC and lake water quality, and will provide a practical basis for the identification of critical regions for lake.

## 1. Introduction

Lake ecosystems supply essential goods and services to sustain ecosystems and the livelihoods of people living in their watersheds [1,2]. In spite of their critical importance, many lakes in China are approaching their limits in terms of ecological environmental quality and lake eutrophication as a result of land use/cover change (LUCC) [3]. LUCC in the form of urbanization, agricultural land fragmentation, and removal of vegetation has greatly increased the pollution loads from non-point sources (NPS), which in turn gradually increases the nutrient concentrations in lakes and degree of eutrophication in lakes, and finally results in outbreaks of algal blooms [4,5]. The lake water deterioration has an extremely serious effect on the public health of local inhabitants. Therefore, there is a critical need to study the linkage between land use and water quality in lake watersheds for the purpose of aquatic environment protection.

The strong relationship between LUCC and water quality at the watershed scale has been widely demonstrated by various studies [6,7]. Lake degradation, by way of non-point source pollution, results from conversion of land uses from native cover to agriculture and urban uses [8]. Specifically, the nutrient levels including total nitrogen (TN), total phosphorus (TP) and chlorophyll would be increased as a function of declining original forestland and grassland. Therefore, the original forestland, arable land and urban land have been identified as the land uses most sensitive to lake quality changes. In addition, the land fragmentation, excessive fertilization and artificial forest harvesting make major contributions to the increase of nutrient loads in inflowing rivers, which would directly increase the extent of lake eutrophication [9]. This cognition indicates that not only the changes of land use type proportion, but also the differential spatial and landscape characteristics constitute key contributing factors for lake water quality [10], and a reasonable land use adjustment and allocation is useful for lake water resources protection [11]. Therefore, several recent studies have focused on the spatial division of watershed environment governance using spatial analysis, statistical analysis and hydrologic modeling, in which the choosing of an appropriate spatial observational unit is critical [12]. Additionally, most studies have considered the relationship between land use and water quality by using segmented sub-watersheds [13]. However, the dense river network is close-knit spatially in some plain areas, which results in the difficulty in differentiating sub-basins [14]. For these cases, another possible approach is to construct a series of buffer rings around lakes using GIS-based proximity modeling [15]. However, this research method has rarely been used in the typical plain region of China, and previous studies on multi-buffer spatial units have frequently obtained inconsistent results. For instance, some studies have shown that LUCC indices can explain more information on lake water quality variables for buffer regions closer to lakes [16]. Conversely, other studies have indicated that the land characteristics at the whole-catchment scale had a greater influence on the water than the characteristics of the 100 m buffer ring [17], some studies have even showed that the relationship between lake and land use was enhanced at first and then weakened as the distance reached a certain zone [18]. The inconsistent results were attributed to a variety of factors. Firstly, single time phase was less suitable than multiple time sequence [19]. More importantly, inconsistent lake water quality indices were used in the previous studies. As shown above, a variety of water indicators including TP, TN, water transparency and phytoplankton composition have been described and correlated with LUCC, and the inconsistent indicators always resulted in different assessment results [20]. For this case, a comprehensive and unified lake water indicator is necessary.

Taihu Lake is well known as a plain lake that has suffered from severe eutrophication over the past two decades. In the meantime, drastic land use/cover adjustments have also occurred during this time period [21]. The Water Resources Protection Bureau of Taihu Basin has designated the 5 km area around the lake as a critical lake conservation region, which provides the practical basis for the identification of the critical lake conservation region and lake management [22]. However, how to designate the critical watershed region more reasonably based on LUCC analysis, and whether the critical watershed regions are differentiated among different lake division. These issues are need to be extensively investigated. Therefore, in this study, we selected several typical districts within Taihu Lake as study areas. A comprehensive lake quality indicator which is effective in the characterization of lake eutrophication extent was proposed to correlated with LUCC indicators in multi-buffer-ring regions. The overall objectives of this paper were: (1) to identify the critical LUCC indicators influencing lake eutrophication in each buffer region and lake districts; (2) to identify the most sensitive buffer region showing the greatest relationship to lake eutrophication, and discuss the possible differences among different lake divisions.

## 2. Material and Methods

### 2.1. Study Site

Taihu Lake, with an area of 2338.1 km^2^, is one of the five largest freshwater lakes in China. The drainage area of the lake includes Shanghai City, Jiangsu Province, and Zhejiang Province. As a typical Chinese urban-rural area, the Taihu Lake watershed has experienced significant urban expansion in the last 20 years. Unplanned urbanization, deforestation and soil erosion have resulted in a heavy inflow of nutrients into the lake from catchment areas. As a result, Taihu Lake has become one of the most seriously eutrophied lakes in China [23].

The existing studies always divided the whole lake into several sub lake basins due to the fact the whole lake catchment area is enormous as well as the correspond catchment region across three different provinces which were processed with various LUCC status. Three typical lake divisions including Mei Liang Lake (ML), Zhu Shan Lake (ZS) and the Western Coastal region (WC) have experienced the most severe eutrophication status [24]. Moreover, the three divisions also belong to the inlets of Taihu Lake, which is greatly affected by LUCC in the catchment area. The catchment area of the three divisions includes Yixing, Wujin, and part of Wuxi city, which has historically been covered by a large area of agricultural land. In the recent 20 years, this region has witnessed rapid urbanization expansion and population growth, which have not only encroached the forestland and agricultural land, but also aggravate the industrial point sources and agricultural non-point source pollution, even resulting in suspended matter concentration and lake water degradation [25,26].

**Figure 1:**
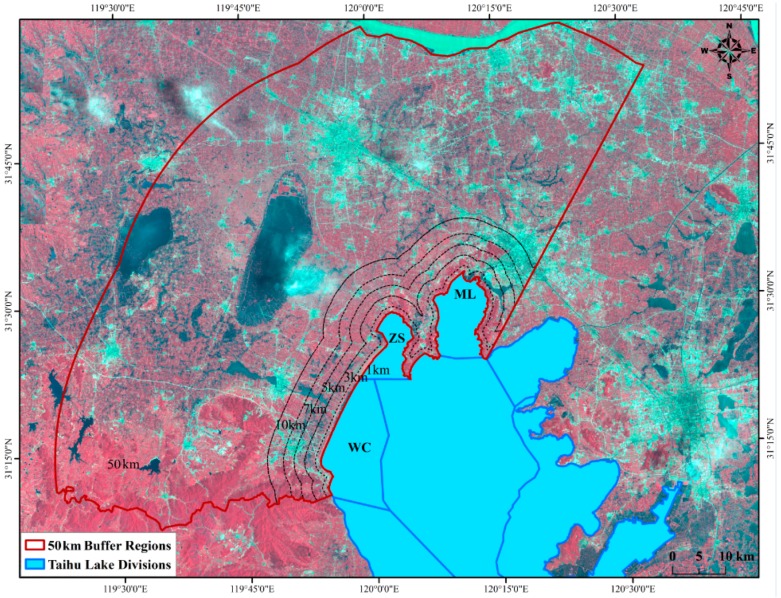
The location of study site and three lake divisions.

Therefore, we selected the three typical lake divisions as the study sites. In addition, in order to compare the relationship between LUCC and lake water degradation on a buffer distances scale and catchment scale, and thereby to identify the most sensitive watershed region, six buffer rings which represented different distances to each lake division boundary were defined and represented spatially (Figure 1). The different distance of each buffer region were including 1 km (B1), 3 km (B2), 5 km (B3), 7 km (B4), 10 km (B5) and 50 km (B6). Moreover, the location of B6 region is proximity to the whole Taihu Lake catchment boundary, which could be represented as the catchment scale to a great extent [25]. After all, the typical lake divisions and selected catchment regions were defined and represented spatially by ArcGIS 10.0 (ESRI, California, USA)

### 2.2. Land use/cover change *(*LUCC*)* Indices

The LUCC indices include the land use change data and the corresponding landscape characteristics. To assess changes in land use, Landsat Thematic Mapper (TM) satellite images covering the study area were downloaded from U. S. Geological Survey [27]. The TM images consisting of six multispectral bands at a spatial resolution of 30 m were acquired for each October in 1990, 1995, 2000, 2002, 2005, 2007, 2010, and 2012. All the data were downloaded when satisfying the selection criteria that cloud percentage were under 10%, finally totally eight images covered the whole temporal sequence were obtained. Before the classification, the data preprocessing such as atmospheric correction and geometric correction were conducted by ENVI 5.0. Then object oriented interpretation was carried out to create land cover maps. Six land cover types were recognized: (1) original forest (OF); (2) planted forest (PF); (3) agricultural land (AL); (4) construction land (CL); (5) wetland (WL); (6) unutilized land (UL). The interpretation algorithm and mode were with reference to land use classification datasat of Jiangsu Province, China for 2000, 2005, and 2010, respectively. These data products were produced by the Chinese Academy of Science Data Center for Geography and Limnology Science (Nanjing, China), and the validation of interpretation results were conducted by fieldwork (75 samples), the data accuracy reached 85%. Finally, spatial data on land use change were acquired for the period 1990–2012. The variation trends in the proportion of each land use type were calculated as land use indices.

To assess the changes in landscape characteristics over temporal sequence and each buffer regions, some typical landscape indices that reflected the general spatial structural features of designated regions were selected and measured. The indices selected were Shannon diversity index (SHDI), Shannon evenness (SHEI), fragmentation index (*Ci*), and the mean patch fractal dimension (FRAC). Specially, SHDI was applied to measure the land use diversity in certain areas, in which the value proportionally increases with the number of different land use types increases; SHEI expresses the even distribution of area among land uses, the value approaches “1” when the distribution of area becomes increasingly even; FRAC is an index characterizing fractal patterns by quantifying the area complexity as a ratio, the value approaches to “1” when certain patch pattern is structured linearly; Whereas, the *Ci* was used to quantifying the spatial structure complexity of internal landscape, the value increases when the complexity is aggravated. All the landscape indices were measured by using FRAGSTATS 3.3 [28].

### 2.3. Extraction of Algal Area from Remote Sensing Images

A comprehensive target was used to explain water quality instead of the inconsistent indices used in previous studies. Taihu Lake is a typical eutrophication lake in China, where the algae bloom phenomenon is generated due to the action of several physical, biological and chemical indicators. Therefore, in this study, the algae area characteristic was used as a comprehensive indicator to establish the correlation with LUCC.

The algae area characteristic can spatially represent the lake eutrophication status in an effective way. It can also be assessed by remote sense satellite images. Some conventional remote sensing indicators including Normalized Difference Vegetation Index (NDVI) and Enhanced Vegetation Index (EVI) were used in previous studies. However, these indices showed some inevitable limitations in algae mapping as algae and aquatic vegetation pixels were difficult to distinguish, which overestimates the actual algal bloom area. Therefore, Hu proposed a Floating Algae Index (FAI) in 2009 [29], which is calculated by spectral bands including NIR, SWIR, and RED based on moderate-resolution imaging spectroradiometer (MODIS) images. This method is more accurate for algae pixel identification. The FAI is defined as Equations 1 and 2.

(1) $$FAI=R_{rc}\left(859\right)-{R^{\prime }}_{rc}(859)$$

(2) $${R^{\prime }}_{rc}\left(859\right)=R_{rc}\left(645\right)+\left[R_{rc}\left(1240\right)-R_{rc}\left(645\right)\right](859-645)/(1240-645)$$

where $R_{rc}\left(\lambda \right)$ the baseline reflectance of each band is corrected using the Rayleigh scattering method. According to Hu’s results, the threshold of −0.004 could be designated as the distinction criteria of algae and non-algae pixel [30].

For this case, MODIS images downloaded from National Aeronautics and Space Administration [31] were used to extract the algae area from 2000–2012 according to FAI index. In additional, it is worth mentioning that MODIS images could not be acquired until the year 2000*,* the choice of RVI which represented by band 4/band 3 was designated as a substitute. The model could be implemented with Landsat TM images. In the RVI model, band 4 represents the reflectance of NIR band, and band 3 represents the reflectance of the RED band, the threshold of 1 was designated as the distinction criteria of algae pixel and non-algae pixel. This method had been achieved ideal effect in relative studies focused on Taihu Lake [32].

According to FAI and RVI, the algae mapping should be conducted in eight years including 1990, 1995, 2000, 2002, 2005, 2007, 2010 and 2012. From the basis that the spatial variability of algae area changes greatly in a single year, the algae area was mapped for each month of the year under the premise of the remote sensing image that could be captured and satisfying the criteria about cloud coverage percentage below 10%. Moreover, in order to ensure that the algae data and land use indices could be associated in temporal scale, the averaged results for each month per year were designated as the value of that year. Moreover, three lake divisions were also considered respectively.

### 2.4. The Relationship between Land Use/Cover Change *(*LUCC*)* and Algae Area

To provide a foundation for the analysis of the relationship between LUCC indices and algal area over the time period, the algal area extracted from remote sensing images of each lake division was modeled using the LUCC indices. From previous studies, the exponential model can explain the relationship between water quality and LUCC indices more effectively than other models. Therefore, the exponential model would be used in this study and expressed as Equation 3.

*A_algae_ = a × Exp* (*b1 × P_of_ + b2 × P_pf_ + b3 × P_al_ + … + b9 × P_Ci_ + b10 × P_FRAC_*) (3)

where *a* is constants, *A_algae_* reflected the algae area within different lake divisions in the whole temporal sequence, Pi reflected the value of ten LUCC indices including proportions of different land use types and landscape indices, in which the *P_of_* means the proportion of original forestland (OL), and the *P_Ci_* is the value of *Ci* in each buffer regions within. Additionally, *b1* *…* *b10* are coefficients that depict the direction and strength of the relationships between LUCC indices and algae area.

The analysis was carried out by stepwise regression analysis in SPSS 17.0 for Windows.The *F* test was introduced to the stepping method criteria. More specifically, the probability of *F* to be considered significant was < 0.100, and the *t* test was used to identify the critical independent variables (*p* < 0.05) entering in the final stepwise model [33,34]. The modeling was carried out for the three divisions incorporating eighteen buffers, thus providing a basis for identification of the critical LUCC indices and sensitive buffer regions most closely related to algal area in each division.

## 3. Results

### 3.1. Changes in Land Use*/*Cover change *(*LUCC*)* over 20 Years

**Figure 2:**
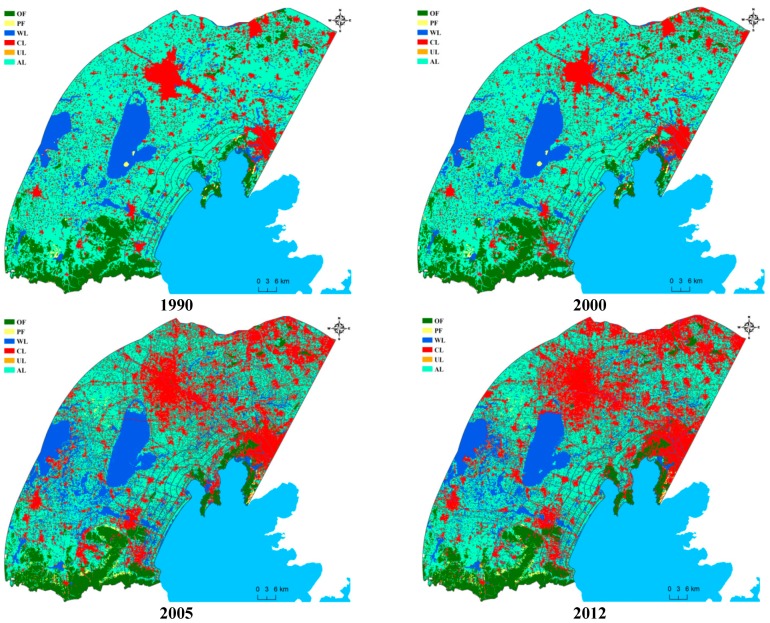
The tendency of Land Use/Cover change (LUCC) over of each buffer regions since 1990–2012.

Six typical land use types of the three lake divisions were classified for the whole temporal sequence, and the spatial distribution tendency of each land use type is shown in Figure 2 (only the maps for 1990, 2000, 2005, and 2012 are shown due to paper size), and the statistic data of each land use proportions were showed in Table 1. With respect to the complete study site reflecting 50 km buffer regions, agricultural land was dominant before 2000, while construction land was mainly present in the northeastern part of ZS region. Since 2000, the area of construction land has increased rapidly in the western part of the WC region, and gradually expanded over the whole area, accompanied by decrease and fragmentation of agricultural land (Figure 2).

In 2012, construction land and agricultural land covered most of the region; the proportions of these two types exceeded 75% in ML (Table 1). In addition, the areas of wetland and original forestland were the third and fourth largest among all land use types. The original forest and planted forest increased since 2000, especially in the region close to Taihu Lake within WC and ML region. The wetland was distributed in ZS more widely than the other two regions in which the proportions were kept around between 18%–20%. More importantly, the change tendencies of OL and WL decreased slightly in full temporal sequence and were mainly reflected in ML and ZS. For instance, the WL proportion in ML decreased from 6.33 to 6.28 within the whole period. Similarly, the OL proportion in ZS showed the similar tendency (Table 1).

**Figure 3:**
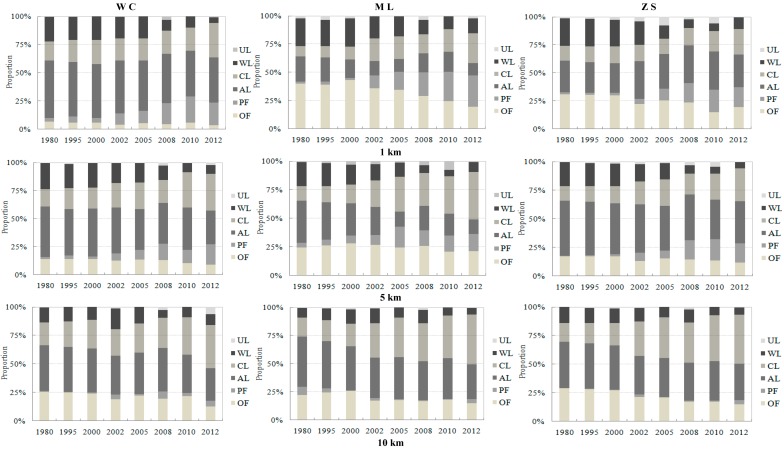
Proportions change tendency of different land use type classified by various buffer distances for three lake divisions.

The LUCC characteristics also varied notably among particular sub-buffer regions, and the proportions of land uses in six buffer zones along each lake division were generated in Figure 3 (only the results of B1 (1 km), B3 (5 km) and B5 (10 km) were listed due to paper size). The agriculture land reduction and Construction land increase tendency seems straightforward, while the most significantly sub-buffer region was not similar within three catchments, in which this tendency was most notable in 1 km and 5 km for ML region, and then became less marked as the distance increased to 10 km. Otherwise, it is need to note that the change of WL were also notable within each catchments and sub-buffer basins, unless the proportion of WL was lower than CL and AL.

**Table 1:** Statistical data of each land use proportion within the full temporal series.

Region	Year	OF %	PF %	AL %	CL %	WL %	UL %
WC	1980	27.49	1.02	41.02	17.02	13.40	0.05
	1995	27.72	1.08	39.51	19.29	11.25	1.15
	2000	26.95	1.14	40.01	18.56	12.59	0.75
	2002	23.94	4.55	34.87	22.12	14.22	0.30
	2005	22.08	1.51	34.63	13.34	15.22	13.22
	2008	22.50	6.41	37.46	15.75	7.73	10.15
	2010	21.87	2.97	39.22	16.52	9.86	9.56
	2012	16.45	5.21	34.38	22.93	10.33	10.70
ML	1980	18.14	3.21	46.54	22.30	6.33	3.48
	1995	19.33	4.88	43.90	22.60	8.47	0.81
	2000	20.73	1.55	42.25	23.91	10.62	0.94
	2002	18.65	2.41	37.92	30.75	8.33	1.94
	2005	17.95	1.65	35.26	37.38	5.95	1.80
	2008	16.76	1.85	33.41	36.11	9.68	2.19
	2010	15.94	1.62	35.33	39.90	6.11	2.10
	2012	15.78	2.64	30.16	44.97	6.28	0.16
ZS	1980	13.79	0.66	39.36	16.24	20.89	9.06
	1995	14.00	0.72	37.57	17.87	22.19	7.66
	2000	12.21	0.79	33.77	24.49	22.48	6.26
	2002	11.02	2.82	28.68	28.68	23.13	5.67
	2005	12.58	0.75	26.98	30.67	19.89	9.13
	2008	11.55	0.98	27.92	31.08	18.39	10.08
	2010	10.33	0.76	26.55	34.30	17.16	10.90
	2012	11.00	3.79	25.66	36.09	18.39	5.07

In addition to the changes of different land use type area, the spatial distribution and landscape features showed significant changes during the whole period. The change tendencies of SHDI, SHEI, and *Ci* at the land level are shown in Table 2. Firstly, the SHDI and SHEI indices for the three divisions are similar, and show an increasing tendency over 20 years. However, this tendency is non-linear, and the years 2002 and 2008 seem to be inflection points. For instance, as is shown in the ML region, the SHDI value of the 1 km region increased until 2002 and between 2005 and 2008. The maximum values appeared in 2002 (1.522) and 2008 (1.605), which were significantly higher than in other years.

This tendency is also apparent in other regions and buffers. Secondly, the 1 km region owned the severest fragmentation degree in three divisions. The *Ci* index decreased to some extent as the distance to lake increased. The WC region processed with the severest fragmentation degree in which the *Ci* was higher than 10 since 2002 and even reached 22.955 in 2012, which was well above the levels of ML and ZS. In addition, the *Ci* showed an increasing tendency over the whole time period, while the growth tendency has slowed down since 2008. Thirdly, the fractal dimensions were consistent in three regions, which did not show significant differences among different regions and different years.

**Table 2:** Change tendencies of landscape indices among different buffer regions.

Region	Year	B1 (1 km)				B3 (5 km)				B5 (10 km)			
		SHDI	SHEI	*Ci*	FRAC	SHDI	SHEI	*Ci*	FRAC	SHDI	SHEI	*Ci*	FRAC
WC	1980	1.079	0.715	6.558	1.0724	1.269	0.725	2.691	1.0684	1.296	0.699	2.049	1.0548
	1995	1.112	0.737	6.153	1.0625	1.295	0.737	2.610	1.0796	1.341	0.743	2.008	1.0684
	2000	1.183	0.735	12.677	1.0898	1.252	0.778	3.915	1.0783	1.311	0.732	2.661	1.0645
	2002	1.427	0.797	15.382	1.0373	1.409	0.820	4.456	1.0349	1.523	0.850	2.931	1.0347
	2005	1.190	0.740	12.137	1.0988	1.308	0.813	3.807	1.0775	1.360	0.759	2.606	1.0663
	2008	1.492	0.833	17.005	1.0397	1.429	0.837	5.290	1.0375	1.567	0.875	3.093	1.0388
	2010	1.042	0.648	16.870	1.087	1.329	0.757	5.329	1.0752	1.297	0.724	3.026	1.062
	2012	1.277	0.713	22.955	1.1173	1.455	0.812	5.770	1.0348	1.395	0.867	3.088	1.0328
ML	1980	1.266	0.786	2.975	1.0427	1.375	0.732	1.785	1.0436	1.285	0.756	1.524	1.0254
	1995	1.294	0.815	2.832	1.0578	1.435	0.783	1.699	1.0874	1.348	0.793	1.451	1.0545
	2000	1.288	0.801	3.498	1.0654	1.411	0.787	2.099	1.0623	1.317	0.735	1.792	1.0613
	2002	1.522	0.946	4.046	1.0418	1.588	0.886	2.427	1.0358	1.531	0.855	2.072	1.0338
	2005	1.240	0.770	6.640	1.0678	1.376	0.855	3.084	1.0492	1.316	0.735	3.401	1.0388
	2008	1.605	0.896	7.925	1.0433	1.635	0.913	4.455	1.0417	1.588	0.886	3.803	1.0403
	2010	1.230	0.764	8.448	1.0689	1.327	0.824	4.069	1.0471	1.278	0.713	3.327	1.037
	2012	1.466	0.911	8.257	1.0415	1.469	0.820	4.154	1.0363	1.401	0.782	3.254	1.0325
ZS	1980	1.295	0.695	3.209	1.0145	1.201	0.755	1.925	1.0452	0.816	0.602	1.605	1.0351
	1995	1.265	0.687	3.606	1.0257	1.235	0.812	2.164	1.0388	0.976	0.599	1.803	1.0537
	2000	1.316	0.734	5.654	1.0484	1.164	0.650	3.392	1.0587	0.944	0.527	2.827	1.0163
	2002	1.579	0.881	5.165	1.0422	1.513	0.844	3.099	1.0317	1.367	0.763	2.583	1.0353
	2005	1.330	0.959	5.104	1.0598	1.261	0.910	3.062	1.0458	1.108	0.799	2.552	1.0536
	2008	1.527	0.852	5.929	1.0785	1.388	0.774	3.557	1.0453	1.238	0.691	3.765	1.0721
	2010	1.321	0.953	6.235	1.0964	1.190	0.859	3.141	1.0564	1.071	0.773	3.117	1.0987
	2012	1.403	0.878	6.108	1.1025	1.476	0.824	3.065	1.0324	1.365	0.762	3.154	1.1163

### 3.2. Changes in Algae Area over 20 years

**Figure 4:**
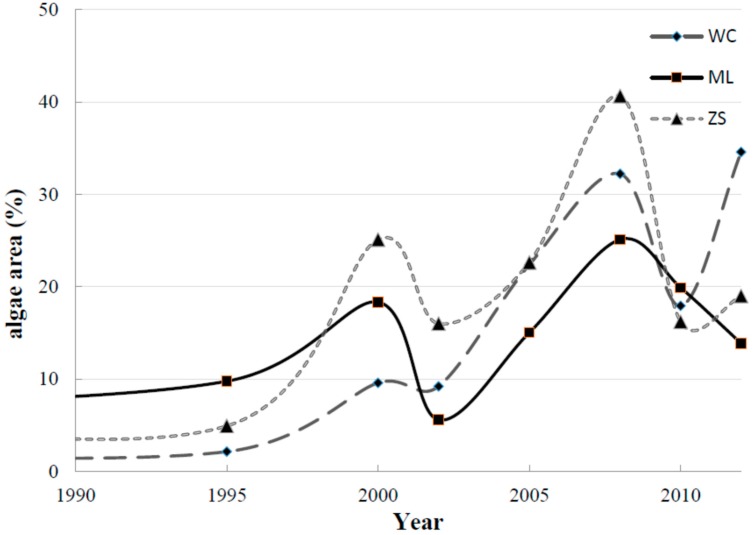
Change tendency of algae area within the three lake divisions since 1990–2012.

From the FAI algorithm, the statistical data of algae covered area among three lake divisions over the 20 years were obtained in Figure 4. In general, the phenomenon of algae bloom was relatively not significant until 2000 and since then increased notably. A non-linear increasing tendency is remarkable in each division (Figure 4). Specially, the algal area was mostly lower than 20% in 2000 except for ZS, while the explosive growth of algal area took place between 2002 and 2008, even reached a peak in 2007 within all the three divisions. Meanwhile, in spite of the decrease in proportions to some extent since 2008, the general algae area was still covered more than the period before 2002. With respect to different divisions, the algal area of ZS was larger than those of ML and WC, in which the algae area exceeded 40% in 2007. In additional, within the other two divisions, ML had a larger algae-covered area than WC until 2000. Since then, the algal area of WC increased rapidly and exceeded the area of ML since 2000, which reached the highest algae-covered proportion in 2012 (34.59%).

### 3.3. Relationship between Land Use/Cover change and (LUCC) Algae areas

The relationships between LUCC and algal area as three divisions considered together were presented firstly in order to analyze the general influence tendency among different distances to lake. *R*^2^ and RMSE were used to assess the models (Table 3).

**Table 3:** Modeling result of LUCC indices and algae area as three divisions combined.

Buffer Region	Distance to Lake	*n*	*R*^2^	RMSE	Significant Factors	
					Land Use	Landscape
B1	1 km	24	0.44	6.78	WL	SHDI
B2	3 km	24	0.66	3.73	WL, PF	SHDI
B3	5 km	24	0.58	8.21	WL, AL	--
B4	7 km	24	0.52	8.39	AL	*C_i_*
B5	10 km	24	0.50	7.53	AL, WL	*C_i_*
B6	50 km	24	0.23	9.04	--	*C_i_*

**Figure 5:**
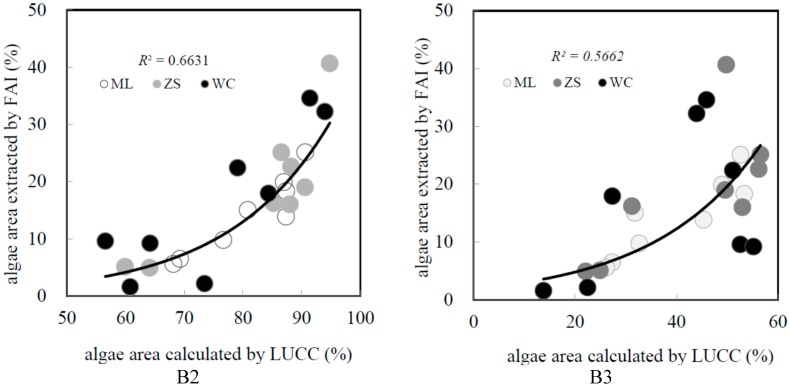
Scatter plots of algal area calculated by LUCC and algal area extracted by FAI for the B2 and B3 buffer regions.

The significant factors that were selected for the modeling process varied as the distance to lake increased. In particular, the land use type wetland (WL) and the landscape index such as SHDI were the critical factors at 1 km and 3 km. As the distance increased, agricultural land and construction land played a critical role in algae area modeling, as well as the landscape index (*Ci*). To assess the model, the B2 (3 km) region showed the best fitting results among five regions and followed by B3 (5 km), in which the *R*^2^ values were 0.66 and 0.58, respectively. Moreover, the *R*^2^ degraded since the buffer distance exceeded 10 km. For example, the *R*^2^ of B6 (50 km) was only 0.23. In other words, the linkage between LUCC and lake water quality was reflected notably in the sub-buffer distance scale but not the whole catchment scale. However, it is important to note that the overall modeling effect was not ideal, in which the *R*^2^ of B2 and B3 were lower than 0.7, and the *R*^2^ were even kept around 0.5 in other regions. To deeply analyze the phenomenon, the scatter plots figure showing the algal area extracted by FAI and that calculated from LUCC indices within B2 and B3 region is shown in Figure 5.

From Figure 5, the most marked characteristic is that the plots for WC region are generally separated from the trend line, while the plots for ML and ZS region are closer to the trend line, from which it could be inferred that the modeling ability of the three regions considered together was degraded due to the influence of WC region. The modeling statistical data for each division are shown separately in Table 4. The distribution of *R*^2^ showed obvious differences among the three lake divisions. Specially, the best-fitted result appeared in B2 for the ML region and in B3 for ZS, in which the *R*^2^ values reached 0.89 and 0.90, respectively. Moreover, *R*^2^ was larger than 0.8 in three buffer regions from B2 to B4 within ML, and a similar situation existed in ZS, in which B2 and B3 showed the optimum *R*^2^ values. Conversely, the fitting ability was poor in WC region, which only showed a relatively good *R*^2^ of 0.67 within the B1 region, and the *R*^2^ values decreased notably as the distance to the lake increased.

**Table 4:** Modeling result of LUCC and algae area as three divisions considered separately.

Buffer Region	B1		B2		B3		B4		B5		B6	
	*R*^2^	Factor	*R*^2^	Factor	*R*^2^	Factor	*R*^2^	Factor	*R*^2^	Factor	*R*^2^	Factor
WC	0.67	WL	0.59	WL	0.42	AL, WL	0.43	AL	0.38	*Ci*, SHDI	0.23	AL, *Ci*
ML	0.59	WL, SHDI	0.89	WL, SHDI	0.85	WL, AL, *Ci*	0.80	AL, WL, *Ci*	0.60	*Ci*	0.31	*Ci*
ZS	0.48	WL	0.82	AL, WL, SHDI	0.90	AL, SHDI, *Ci*	0.55	*Ci*	0.60	AL, *Ci*	0.20	AL, *Ci*

For all the above, the LUCC within ML and ZS region were more closely related to algal area than in WC region. Moreover, the relationship was more closely tied in the buffer scale rather than in whole catchment scale, and mainly reflected in B2 (3 km) and B3 (5 km) region.

## 4. Discussion

The above results showed that the LUCC plays a critical role in lake degradation. However, the relationship showed some differences among different lake divisions and corresponding watersheds. Elucidation of the intrinsic mechanism is critical for watershed management and LUCC adjustment. Therefore, the differences in relationship between LUCC and lake algal blooms among different divisions and buffer distances will be discussed for two aspects: the critical LUCC indices and the most sensitive watershed region.

### 4.1. Critical Land Use/Cover change *(*LUCC*)* Indices Affecting Lake Algal Blooms in Different Lake Divisions

**Figure 6:**
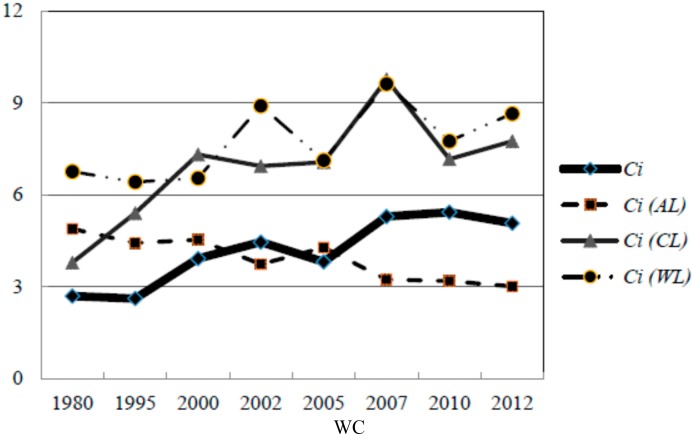

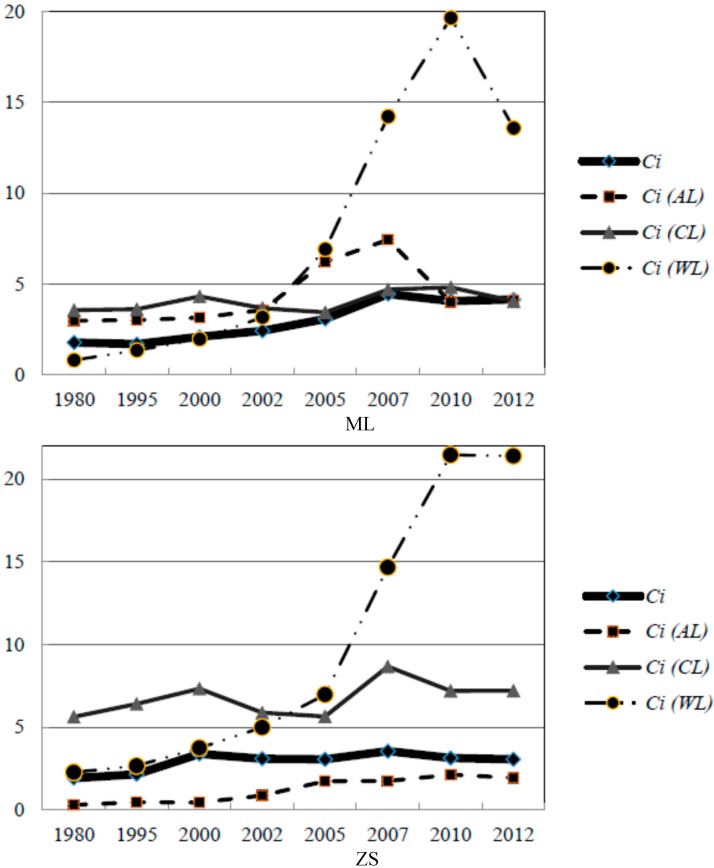
Change tendency of *Ci* in land level and in land class level.

The critical LUCC indices affecting lake algal blooms varied as the buffer distance to the lake changed. Considering the three divisions as an entirely configuration, Table 2 shows that the land use type wetland (WL) and the landscape index such as SHDI were the critical factors in for 1 km and 3 km buffer regions, even the WL was also critical factor in the 5 km region. As the distance increased, the *Ci* showed increasing importance on the lake quality. Considering each lake division separately, the critical factors were similar for the three divisions, which was also consistent with the results shown in Table 3. Some noteworthy features are:

(1) The land use types including wetland and agricultural land, accordance with the landscape indices including SHDI and *Ci* appeared to be the most significant factors from Table 3 and Table 4. In particular, the WL and SHDI related to lake algae bloom more closely when the buffer distance was proximity to lake water. Importantly, the rapid urbanization process in Taihu basin, the LUCC studies always focused on agriculture land and construction land, and rarely mentioned the role of wetland areas. It is widely acknowledged that the wetlands showed an effective retention ability for nitrogen and phosphorus pollutants in drainage and is favorable for lake water purification and eutrophication inhibition [35]. Therefore, it can be inferred that the wetland plays the most important role in hydrology conditions regulation among various land use types. Actually, the relative studies have been indicated that the impact of hydrology on lake quality is also critical as well as LUCC [36]. For this case, the lake environmental protective effect of wetland should be consider conscientiously in the researches focused on the relationship between LUCC and lake quality.

(2) Beside the area proportion of land use patterns, their spatial distribution exerted a great influence on lake quality. Many related studies have confirmed that the agricultural lands which were distributed in a scattered way, were more likely to generate non-point source pollution (NPS) than the agricultural lands which were structured and contiguous. This recognition corresponds to the critical indicators in landscape theory including Shannon diversity index (SHDI), Fragmentation index (*Ci*) and Fractal dimension index, in which the *Ci* showed the greatest adversely effect on lake quality [37,38]. As shown in Table 3 and Table 4, it can be inferred that *Ci* was the critical factor from 5 km to 50 km region in ML and ZS regions, while the SHDI seems to be the critical factor from 1 km to 5 km. This is due to the close distance to the lake with smaller buffer regions. The importance of the fragmentation degree could not be seen effectively in a relatively small area [39]. Conversely, SHDI appeared to be the critical factor in the buffer regions between 1 km and 5 km, which confirmed the recognition that the homogeneity of land use type was beneficial for the control and management of algal blooms [40].

(3) Agriculture land and construction land greatly influenced lake water quality, and were particularly reflected in the buffer regions from 5 km to 50 km. In other words, their importance was not as significant as in previous studies where the distance to the lake was less than 5 km. These results confirmed that the influence of LUCC on lake quality varied in different distance regions [41,42,43]. It is worth noting that AL becomes a critical factor as the buffer distance expands to 5 km (Table 3). Moreover, AL is also appeared in Table 4 accompanied with the landscape fragmentation index *Ci*. To understand this phenomenon, this study also calculated *Ci* at class level, which allowed analysis of *Ci* for some typical land use types (AL, WL, and CL) among different regions. The variation tendency of *Ci* for each landscape level in the 5 km buffer region is used as a demonstration and shown in Figure 6.

The change tendency for *Ci* in land level was similar to the *Ci* curve of for AL in ML and ZS regions. The ML region exhibited the most consistent tendency between *Ci* at the land level and at AL class level. According to Figure 6, the two curves almost coincide with each other since 2000. This indicates that the degree of fragmentation of agricultural land patches has largely determined the fragmentation degree of the entire region for ML and ZS. Therefore, the impact of agricultural land on lake water quality increases more than 5 km from the lake, and this influence cannot be generalized as a simple linear relation. Specifically, the proportion of AL was negatively correlated with the algal area, indicating that lake water eutrophication will be alleviated to some extent as the AL proportion kept stable or grows when the distance to lake was more than 5 km. Furthermore, under the premise of sufficient agriculture land amount, the spatial arrangement is also equally important, because the fragmentation of AL is positively correlated with the algal extent in the corresponding lake region [44,45].

### 4.2. The Relationship between Land Use/Cover Change *(*LUCC*)* and Algal Blooms for Different Lake Divisions and Buffer regions

The relationships between LUCC and algal blooms varied among the three lake divisions and five buffer distance regions. Put simply, LUCC was poorly related to algal area within the WC region, Conversely, LUCC showed the closest relationship with algal area in 3 km and 5 km buffer regions for ML and ZS regions. First, the impact of LUCC in WC region was not as drastic as the other two regions. As shown in Table 3, the determination coefficients between LUCC indices and algal area were below 0.6, except for B1 region which reached 0.67. The unsatisfactory relationship between LUCC and algae area in WC resulted from two respects: (1) in spite of the fact agricultural land and construction land account for more than 70% of the WC region, the conversion trends of these land use types in the past 20 years were non-significant when compared with the other two regions. Therefore, it can be inferred that LUCC in the WC watershed was the least remarkable among the three watersheds. Nevertheless, the algal area in WC showed notable fluctuations from 1990 to 2012, with an obvious increase between 2002 and 2008, and another between 2010 and 2012 (Figure 4). The entirely different change tendencies revealed that the impact of LUCC in the WC region was non-significant and indirect for lake algal area variation; (2) For the 1 km buffer region in WC, wetland and original forest, which are acknowledged to be typical land use types for pollutant loading interception and purification, and also accounted for the smallest proportion among three sub-watersheds. Moreover, the two land use types in WC were rarely distributed around the lakeshore. This resulted in that the non-point source pollution load (such as nitrogen and phosphorus) in the watershed was prone to affect Taihu Lake eutrophication [46,47]. Therefore, the relationship between LUCC in the 1 km buffer region and lake algal blooms was relatively significant. When the buffer region between 3 km and 50 km was taken into consideration, an increasing tendency in wetland and original forest was notable, and these land use types were also concentrated in terms of their spatial distribution. These features meant that the interception ability of NPS loading was greatly enhanced compared with the 1 km region. In other words, the impact of LUCC on lake water quality was indirect as the buffer distances between 3 km to 50 km.

Second, LUCC was closely related to lake algae area in ML and ZS regions, and the degree of relationship was enhanced at first and then weakened as the distance to lake increased. In other words, the 3 km and 5 km regions seem to be the most sensitive region, but not the whole catchment scale. These results support the conclusions of previous studies which showed that the amount of critical land use type and area was not sufficient when the detected region was less than the effective buffer region, leading to the fact that LUCC was not significantly related to lake water quality. On the other hand, other land use types were needed to intercept into the consideration as the distance to lake beyond the effective buffer region, which would also degraded the impact of LUCC on lake water quality [12]. In our study, wetland (WL) was one of the critical land use pattern within the 5 km region, which plays an important role in pollutant loading interception and purification as well as forestland (OF). Moreover, the proportions of WL and OF within the 5 km buffer region of the ML and ZS region decreased more significantly than the other buffer regions between 1980–2012 (Figure 3). However, this tendency became relatively insignificant as the distance increased due to the construction land covered more and more land area, and even extended into the whole catchment. The construction land and arable land accounted for 75% within ML and 60% within ZS (Table 1). The WL proportion within ML region was only about 6% and remained stable during the full temporal series. These features were severely limited and inhibited the positive effect of wetland and original forestland on lake water quality. Therefore, it can be inferred that 5 km distance, not the whole catchment region, seems to be the inflection point of WL and OF proportion variation, which is consistent with the change tendency for the relationship between LUCC and algal area.

## 5. Conclusions

By using three typical eutrophication lake divisions (ML, ZS, and WC) and their watersheds within the Taihu Lake basin as study sites, this study identifies the sensitive LUCC indices and buffer distance regions that significantly affect lake eutrophication by modeling the algal area of each lake division with LUCC indices.

The impact of LUCC in WC region was not as drastic as the other two regions due to a large area of wetlands and forest zone cover around the lakeshore. In contrast, LUCC within the ML and ZS regions affected the lake algal area more directly, and the wetland (WL) and landscape indexes such as SHDI seems to be the most sensitive factors, even reflected within 5 km region, and the importance of agricultural land (AL), construction land (CL) and landscape fragmentation (*Ci*) were just gradually reflected as the buffer distance to lake divisions increased. However, the most sensitive regions were found in 3 km to 5 km both in ML and ZS regions, rather than the whole catchment. These findings confirm that the comprehensive strategy for water environment management which designated 5 km region along the lake as the critical lake conservation region is reasonable in general, although some typical lake divisions need to be considered separately in the future.

## References

[1] Naiman R.J., Magnuson J.J., McKnight D.M., Stanford J.A. (1995). The Fresh-Water Imperative: A Research Agenda.

[2] Stenseth N.C., Mysterud A., Ottesen J.G., Hurrell W., Chan K.S., Lima M. (2002). Ecological effects of climate fluctuations. Science.

[3] Erol A., Randhir T.O. (2013). Watershed ecosystem modeling of land-use impacts on water quality. Ecol. Model..

[4] Gibson G., Carlson R., Simpson J., Smeltzer E., Gerritson J., Chapra S., Heiskary S., Jones J., Kennedy R. (2000). Nutrient Criteria-Technical Guidance Manual: Lakesand Reservoirs.

[5] Norton L., Elliott J.A., Maberly S.C., May L. (2012). Using models to bridge the gap between land use and algal blooms: An example from the Loweswater Catchment, UK. Environ. Modell. Softw..

[6] Palmer M., Bernhardt E., Chornesky E. (2004). Ecology for a crowded planet. Science.

[7] Jones J.R., Knowlton M.F., Obrecht D.V., Cook E.A. (2004). Importance of landscape variables and morphology on nutrients in Missouri reservoirs. Can. J. Fish. Aquati. Sci..

[8] Tong S.T.Y., Chen W.L. (2002). Modeling the relationship between land-use and surface water quality. J. Environ. Manag..

[9] Wang X. (2001). Integrating water-quality management and land-use planning in a watershed context. J. Environ. Manag..

[10] Amiri B.J., Nakane K. (2009). Modeling the linkage between river water quality and landscape metrics in the Chugoku district of Japan. Water Resour. Manag..

[11] Martinez J.M.A., Seoane S.S., Calabuig E.D.L. (2011). Modelling the risk of land cover change from environmental and socio-economic drivers in heterogeneous and changing landscapes: The role of uncertainty. Landscape Urban Plan..

[12] Guo Q.H., Ma K.M., Zhang Y. (2009). Impact of land use pattern on lake water quality in urban region. Acta Ecol. Sin..

[13] Weller D.E., Jordan T.E., Correll D.L., Liu Z.J. (2003). Effects of land-use change on nutrient discharges from the Patuxent River watershed. Estuaries.

[14] Yang X.J. (2012). An assessment of landscape characteristics affecting estuarine nitrogen loading in an urban watershed. J. Environ. Manag..

[15] Guo Q.H., Ma K.M., Yang L., He K. (2010). Testing a dynamic complex hypothesis in the analysis of land use impact on lake water quality. Water Resour. Manag..

[16] Sawyer J.A., Stewart P.M., Mullen M.M., Simon T.P., Bennett H.H. (2004). Influence of habitat, water quality, and land use on macro-invertebrate and fish assemblages of a southeastern coastal plain watershed, USA. Aquat. Ecosyst. Health.

[17] Sliva L., Williams D.D. (2001). Buffer zone *vs.* whole catchment approaches to studying land-use impact on river water quality. Water Resour..

[18] Hunsaker C.T., Levine D.A. (1995). Hierarchical approaches to the study of water quality in rivers. BioScience.

[19] Hatfield J.L., McMullen L.D., Jones C.S. (2009). Nitrate-nitrogen patterns in the Raccoon River Basin related to agricultural practices. J. soil water conserv..

[20] Chang H. (2008). Spatial analysis of water quality trends in the Han River basin, South Korea. Water Res..

[21] Qin B.Q., Hu W.P., Chen W.M. (2004). Evolution Process and Mechanism of Tai Lake Environment.

[22] Kong F.X., Hu W.P., Fan C.X., Wang S.M., Xue B., Gao J.F., Gu X.H., Li H.P., Huang W.Y., Chen K.N. (2006). Research and strategic thinking for water pollution control and ecological restoration in Taihu Basin. J. Lake Sci..

[23] Zhu G.W. (2008). Eutrophication status and causing factors for a large, shallow and subtropical lake Taihu, China. J. Lake Sci..

[24] Qin B.Q., Zhu G.W., Gao G., Zhang Y.L., Li W., Paerl H.W., Carmichael W.W. (2010). A drinking water crisis in Lake Taihu, China: Linkage to climatic variability and lake management. Environ. Manag..

[25] Yang S.Q., Liu P.W. (2010). Strategy of water pollution prevention in Taihu Lake and its effects analysis. J. Great Lakes Res..

[26] Gao Y.N., Gao J.F. (2010). Delineation of aquatic eco-regions in Taihu lake basin. Geogr. Res..

[27] EarthExplorer. http://earthexplorer.usgs.gov.

[28] McGarigal K., Marks B.J. (1995). FRAGSTATS: Spatial Pattern Analysis Program for Quantifying Landscape Structure.

[29] Hu C.M. (2009). A novel ocean color index to detect floating algae in the global oceans. Remote Sens. Environ..

[30] Hu C.M., Lee Z.P., Ma R.H., Yu K., Li D.Q., Shang S.L. (2010). Moderate resolution imaging spectroradiometer (MODIS) observations of cyanobacteria blooms in Taihu Lake, China. J Geophy. Res..

[31] National Aeronautics and Space Administration. https://modis.gsfc.nasa.gov.

[32] Duan H.T., Zhang S.X., Zhang Y.Z. (2008). Cyanpbacteria bloom monitoring with remote sensing in Lake Taihu. J. Lake Sci..

[33] Basnyat P., Teeter L.D., Lockaby B.G. (1999). Relationships between landscape characteristics and nonpoint source pollution inputs to coastal estuaries. Environ. Manag..

[34] Ferguson C.A., Carvalho L., Scott E.M., Bowman A.W., Kirika A. (2008). Assessing ecological responses to environmental change using statistical models. J. Appl. Ecol..

[35] Coveney M.F., Stites D.L., Lowe E.F., Battoe L.E., Conrow R. (2002). Nutrient removal from eutrophic lake water by wetland filtration. Ecol. Eng..

[36] Jones J.R., Knowlton M.F., Obrecht D.V. (2008). Role of land cover and hydrology in determining nutrients in mid-continent reservoirs: Implications for nutrient criteria and management. Lake Reserv. Manag..

[37] O’Neill R.V., Hunsaker C.T., Jones K.B., Riitters K.H., Wickham J.D., Schwartz P.M., Goodman I.A., Jackson B.L., Baillargeon W.S. (1997). Monitoring environmental quality at the landscape scale. Bioscience.

[38] Alberti M., Booth D., Hill K., Coburn B., Avolio C., Coe S., Spirandelli D. (2007). The impact of urban patterns on aquatic ecosystems: an empirical analysis in Puget lowland sub-basins. Landsc. Urban Plan..

[39] Yang Y.H., Zhou F., Guo H.C., Sheng H., Liu H., Dao X. (2010). Analysis of spatial and temporal water pollution patternsin Lake Dianchi using multivariate statistical methods. Environ Monit. Assess..

[40] Hu J., Liu M.S., Zhou W., Xu C., Yang X.J., Zhang S.W., Wang L. (2011). Correlations between water quality and land use pattern in Taihu Lake basin. Chinese J. of Ecol..

[41] Beaver J.R., Manis E.E., Loftin K.A., Graham J.L., Pollard A.I., Mitchell R.M. (2014). Land use patterns, ecoregion, and microcystin relationships in US lakes and reservoirs: A preliminary evaluation. Harmful Algae.

[42] Deng X., Huang J., Rozelle S., Uchida E. (2008). Growth, population and industrialization, and urban expansion of China. J. Urban Econ..

[43] Fraterrigo J.M., Downing J.A. (2008). The influence of land use on lake nutrients varies with watershed transport capacity. Ecosystems.

[44] Merugu C.S., Seetharaman R. (2013). Comparative analysis of land use and lake water quality in rural and urban zones of south Chennai, India. Environ. Dev. Sustain..

[45] Catherine A., Mouillot D., Maloufi S., Troussellier M., Bernard C. (2013). Projecting the impact of regional land-use change and water management policies on lake water quality: An application to Periurban Lakes and reservoirs. PLoS One..

[46] Nielsen A., Trolle D., Søndergaard M., Lauridsen T.L., Bjerring R., Olesen J.E., Jeppesen E. (2012). Watershed land use effects on lake water quality in Denmark. J. Appl. Ecol..

[47] Vanni M.J., Renwick W.H., Bowling A.M., Horgan M.J., Christian A.D. (2010). Nutrient stoichiometry of linked catchment-lake systems along a gradient of land use. Freshw. Biol..

